# Esmolol increases the fecal abundance of *Lactobacillus* in a rat model of sepsis

**DOI:** 10.1186/s40635-023-00589-1

**Published:** 2024-03-04

**Authors:** Bo Yao, Fu-hua Wang, Xiao-ning Han, Jun Yang, Ping Xue, Qi Qi, Guang-yao Wei, Jin-yan Xing

**Affiliations:** The Department of Critical Care Medicine, The Affiliated Hospital of Qingdao University, Wutaishan Road 1677, Qingdao, 26600 China

**Keywords:** Esmolol, *Lactobacillus*, Sepsis, Colonocyte, Gut microbiota, Nitric oxide

## Abstract

**Background:**

Disorders of the gut microbiome could be responsible for the progression of multiple organ dysfunction syndrome. In this study, we examined the effect of esmolol on the gut microbiome in a rat model of sepsis induced by cecal ligation and puncture (CLP).

**Methods:**

The animals (*n* = 32) were randomly divided into 3 groups: Sham group (sham operation + normal saline treatment, *n* = 8), CLP group (cecal ligation and puncture + normal saline treatment, *n* = 12), and CLP + ESM group (cecal ligation and puncture + esmolol treatment, *n* = 12). After 24 h, feces in the colon were collected for 16S rRNA gene sequencing and nitric oxide analysis. In addition, colon was removed for immunohistochemical staining of inducible nitric oxide synthase (iNOS).

**Results:**

Four rats in the CLP group and two rats in the CLP + ESM group died. The abundance of *Lactobacillus* in the CLP + ESM group was higher than CLP group (*P* = 0.048). In the linear discriminant analysis effect size analysis, *Norank f Muribaculaceae*, *Escherichia–Shigella* and *Lactobacillus* were the predominant bacteria in the Sham group, CLP group and CLP + ESM group, respectively. The iNOS expression in colonocytes stained by brown in the CLP group were much more than Sham group (*P* = 0.001). Compared to CLP group, the iNOS expression in colonocytes reduced after esmolol treatment (*P* = 0.013). The concentration of nitric oxide in colon feces was different in Sham group, CLP group and CLP + ESM group (1.31 ± 0.15μmmol/l vs. 1.98 ± 0.27μmmol/l vs. 1.51 ± 0.14μmmol/l, *P* = 0.001). In addition, the concentration of nitric oxide in CLP group was higher than Sham group (*P* = 0.001) or CLP + ESM group (*P* = 0.001).

**Conclusions:**

Esmolol increased the fecal abundance of *Lactobacillus* in a rat model of sepsis. Moreover, esmolol reduced the iNOS expression of colonocytes and the nitric oxide concentration of colon feces.

## Background

Sepsis results in life-threatening organ dysfunction caused by a dysregulated immune response to infection and is associated with a high morbidity and mortality [1]. The average 30-day sepsis mortality is 24.4%, and the average 30-day mortality for septic shock is 34.7% [2]. The gut microbiome community structure was found to be significantly disrupted in critically ill patients [3]. In addition, the decrease in obligate anaerobes and increase in pathogenic facultative anaerobes were associated with septic complications and mortality [4]. Gut microbiome disruption appears to be an important factor for the development, maintenance, and prognosis of sepsis [5]. The gut microbiome can affect other organ functions through the gut microbiota–brain axis, gut microbiota–lung axis, gut microbiota–heart axis, gut microbiota–kidney axis and so on [6–9]. Therefore, disorders of the gut microbiome could be responsible for the progression of multiple organ dysfunction syndrome [10].

Colonocytes can affect the microenvironment of gut microbiome [11]. C1 colonocytes characterized by elevated synthesis of inducible nitric oxide synthase (iNOS) can affect gut microbes by nitric oxide pathway [12]. Esmolol is a selective β1-adrenergic receptor blocker that is commonly used in the ICU for heart rate control. Esmolol can decrease the inflammation and iNOS expression in sepsis [13]. In a cirrhosis rat model, propranolol, a nonselective β-adrenergic receptor blocker, reduced intestinal bacterial overgrowth and aerobic bacterial stool count [14]. We speculated that esmolol could reduce the iNOS expression of colonocytes and affect the gut microbes. To the best of our knowledge, there have been no studies on the effects of esmolol on the gut microbiome. In this study, we examined the effect of esmolol on the gut microbiome in a rat model of sepsis.

## Materials and methods

### Animals and study design

Male SD rats, 9 weeks of age, 250–300 g of weight and specific pathogen free, were used in this study. The animals were housed with food and water available ad libitum, under a 12-h/12-h light–dark cycle. Experimental protocols were approved by the Institutional Animal Care and Use Committee at our Hospital (QYFY WZLL28147). Experiments were conducted according to the National Institutes of Health Guide for the Care and Use of Laboratory Animals.

The animals (*n* = 32) were randomly divided into 3 groups: Sham group (sham operation + normal saline treatment, *n* = 8), CLP group (cecal ligation and puncture + normal saline treatment, *n* = 12), and CLP + ESM group (cecal ligation and puncture + esmolol treatment, *n* = 12). The sepsis model was established via the cecal ligation and puncture (CLP) method, which was performed in the CLP and CLP + ESM groups, as described previously [15]. After inducing general anesthesia with 1% sodium pentobarbital (40 mg/Kg, intraperitoneal injection), the cecum was ligated at half the distance between the distal pole and the base of the cecum and punctured by 22-gauge needles. After 4 h, rats in the CLP + ESM group were intraperitoneally injected with 40 mg/kg esmolol (Qilu Pharmaceutical Co., Ltd. China). The time point of esmolol injection was determined according to a previous study [13]. The rats in the other groups were administered the same dosage of normal saline. After 24 h, the rats were killed by deep anesthetization with 1% sodium pentobarbital (60 mg/kg, intraperitoneal injection), and decapitated. Colon feces were collected in a sterile collection tube. Within 5 min of collection, feces were placed in a freezer maintained at − 80 °C for subsequent 16S rRNA gene sequencing and nitric oxide measurement. In addition, colon was removed and soaked in 4% paraformaldehyde solution for immunohistochemical staining of iNOS. Average optical density, analyzed with Image-Pro Plus software, was used for semi-quantitative analysis of immunohistochemical staining of iNOS in colonocytes.

### Measurement of nitric oxide in colon feces

1 g colon feces were dissolved by 3 ml phosphate buffer saline. The mixture was then centrifuged and the supernatant was acquired for nitric oxide analysis (Total Nitric Oxide Assay Kit, Beyotime Biotechnology, China. S0023). This Kit firstly reduced nitrate to nitrite by nitrate reductase. Then the nitrite was measured by Griess reagent, and total nitric oxide was estimated.

### 16S rRNA gene sequencing

DNA extraction, 16S rRNA (V3–V4 hypervariable region) amplification, and sequencing were performed according to a previous study [16]. Sequencing was taken on an Illumina MiSeq platform (Illumina) by Majorbio BioPharm Technology Co. Ltd (Shanghai, China). The gut microbiota data were analyzed using QIIME and MOTHUR software. LEfSe cluster or linear discriminant analysis (LDA) was conducted by LEfSe. LEfSe analysis was used to identify the differentially abundant bacterial taxa. LDA scores were used to estimate the effect size of each differentially abundant bacterial taxon. Phylogenetic investigation of communities by reconstruction of unobserved states 2 (PICRUSt 2) was then applied to predict the functional profiles of the gut microbial communities, and the Kruskal–Wallis H test was used to test for differences in the Kyoto Encyclopedia of Genes and Genomes (KEGG) pathways of the gut microbiota. All data were analyzed on the free online Majorbio Cloud Platform (www.majorbio.com).

### Data analysis

The statistical analyses were performed by SPSS software version 22.0 (SPSS, Inc. Chicago, IL, USA) for data of clinical study. In non-normal distribution quantitative data, the results were expressed as median (quartile range). We used the Chi-square test for qualitative data, Mann–Whitney *U* test or one-way analysis of variance to test differences between groups. *P* values < 0.05 were considered as statistically significant.

## Results

### Effect of esmolol on α-diversity and β-diversity

**Fig. 1:**
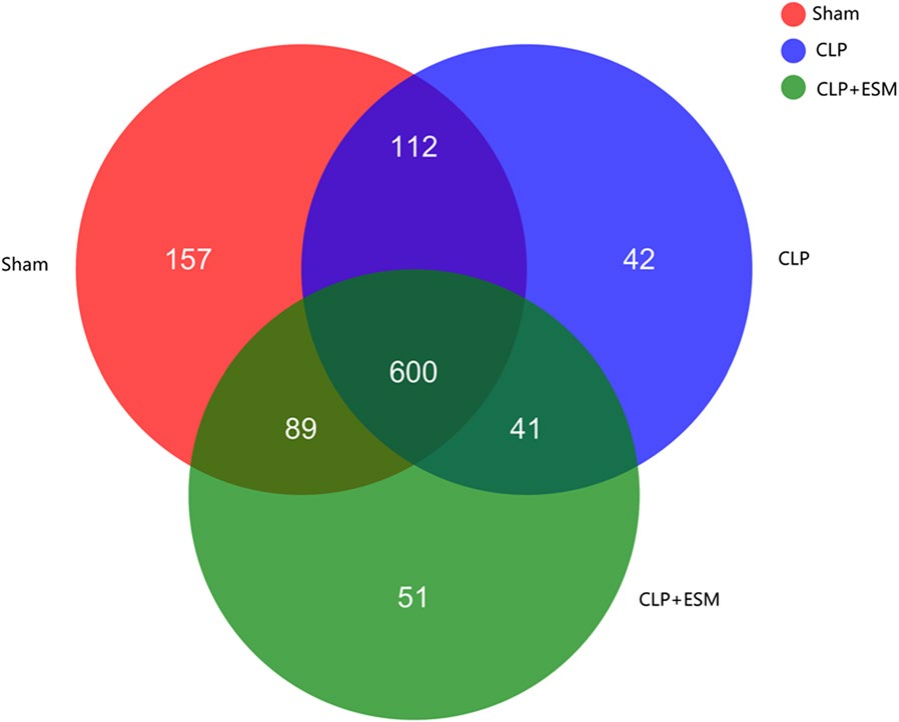
Venn diagram among groups

**Fig. 2:**
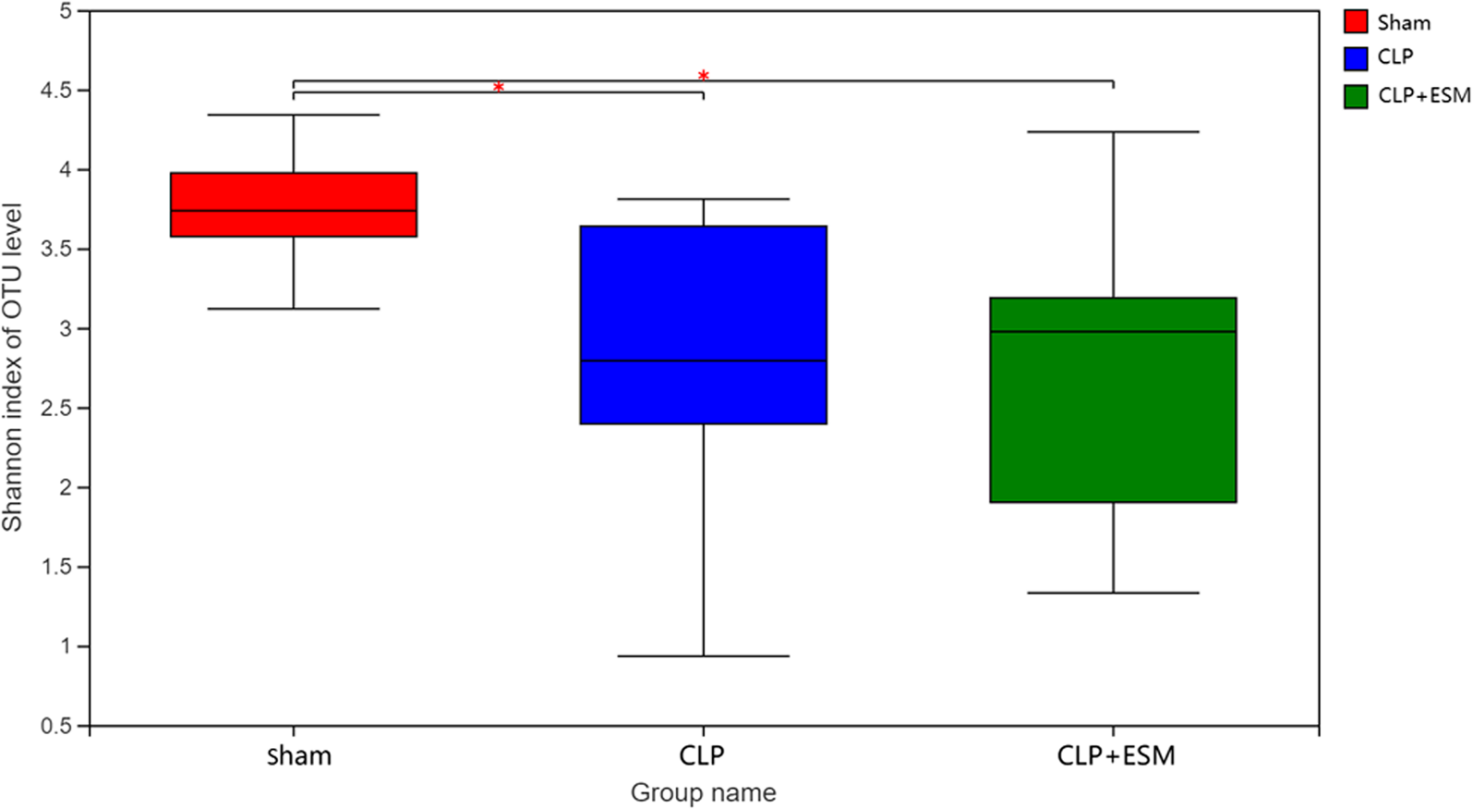
Shannon index among groups (*P* < 0.05 is marked as “*”)

After 24 h, 4 rats and 2 rats in the CLP group and CLP + ESM group, respectively, died. Therefore, the data of 8 rats in the Sham group, 8 rats in the CLP group and 10 rats in the CLP + ESM group were ultimately analyzed. As shown in the Venn diagram (Fig. 1), 958, 795 and 781 operational taxonomic units (OTUs) were obtained, respectively, in the Sham group, CLP group and CLP + ESM group, respectively. Shannon indices are a parameter of α-diversity that are used to indicate sample richness. Our results showed that after CLP treatment, the sample richness of the gut microbiota decreased significantly compared to that of the Sham group [2.743(2.395–3.639) vs. 3.737(3.574–3.974), *P* = 0.031]. However, there was no significant difference in sample richness between the CLP group and the CLP + ESM group [2.743(2.395–3.639) vs. 2.975(1.901–3.188), *P* = 0.999] (Fig. 2).

**Fig. 3:**
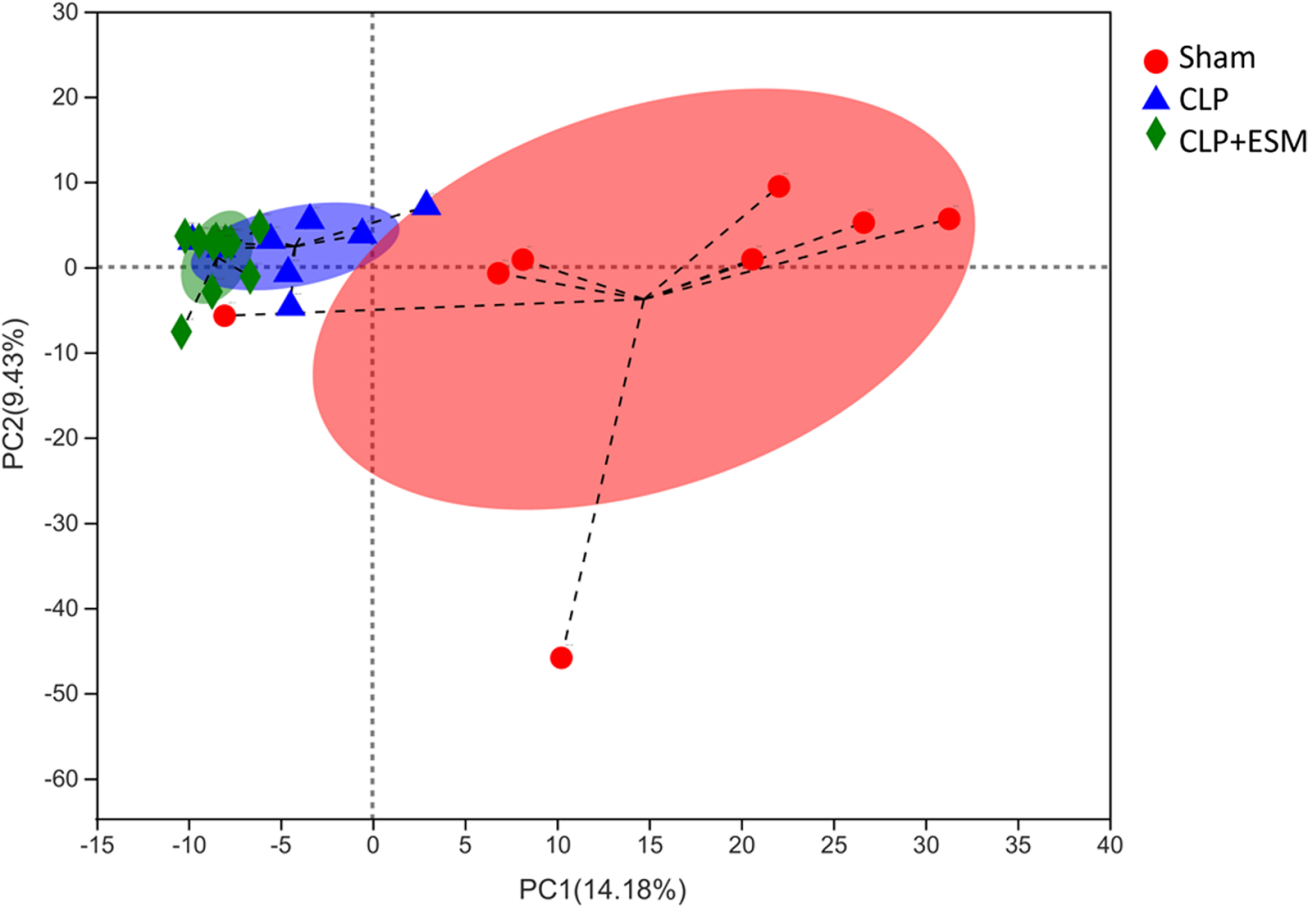
Principal component analysis on operational taxonomic units (OTU) level

Principal component analysis (PCA) is a method used to analyze β-diversity analysis; thus it was used to compare the similarities in microbiota structure among the three groups. The PCA plot revealed the Sham group was significantly different from the CLP group or CLP + ESM group. However, there was no significant difference between CLP group and the CLP + ESM group (Fig. 3).

### Effect of esmolol on bacterial taxonomy

Firmicutes, Bacteroidota, Proteobacteria and Actinobacteria were the four predominant phyla in all groups. The distribution of the bacterial taxa is shown in Table 1. There were significant differences in the abundance proportions of Bacteroidota and Proteobacteria among the sham group, CLP group and CLP + ESM group (33.76% vs. 18.33% vs. 15.23%, *P* = 0.047; 1.93% vs. 32.3% vs. 12.32%, *P* = 0.006). But there was no statistic difference between each pair of the groups (*P* > 0.05) in the abundance proportions of Bacteroidota. The proportion of Proteobacteria was significantly increased after CLP surgery (*P* = 0.01), but there was no significant difference between CLP group and CLP + ESM group in the abundance of Proteobacteria (*P* = 0.07).

**Table 1:** The distribution of main bacterial taxa on phylum level

	Sham group	CLP group	CLP + ESM group	*P* value
Firmicutes (%)	61.37 ± 13.35	42.74 ± 33.34	67.82 ± 30.54	0.148
Bacteroidota (%)	33.76 ± 14.22	18.33 ± 18.67	15.23 ± 17.03	0.047
Proteobacteria (%)	1.93 ± 3.12	32.30 ± 24.73	12.32 ± 16.67	0.006
Actinobacteriota (%)	1.87 ± 1.71	5.85 ± 4.85	4.21 ± 4.31	0.385

**Fig. 4:**
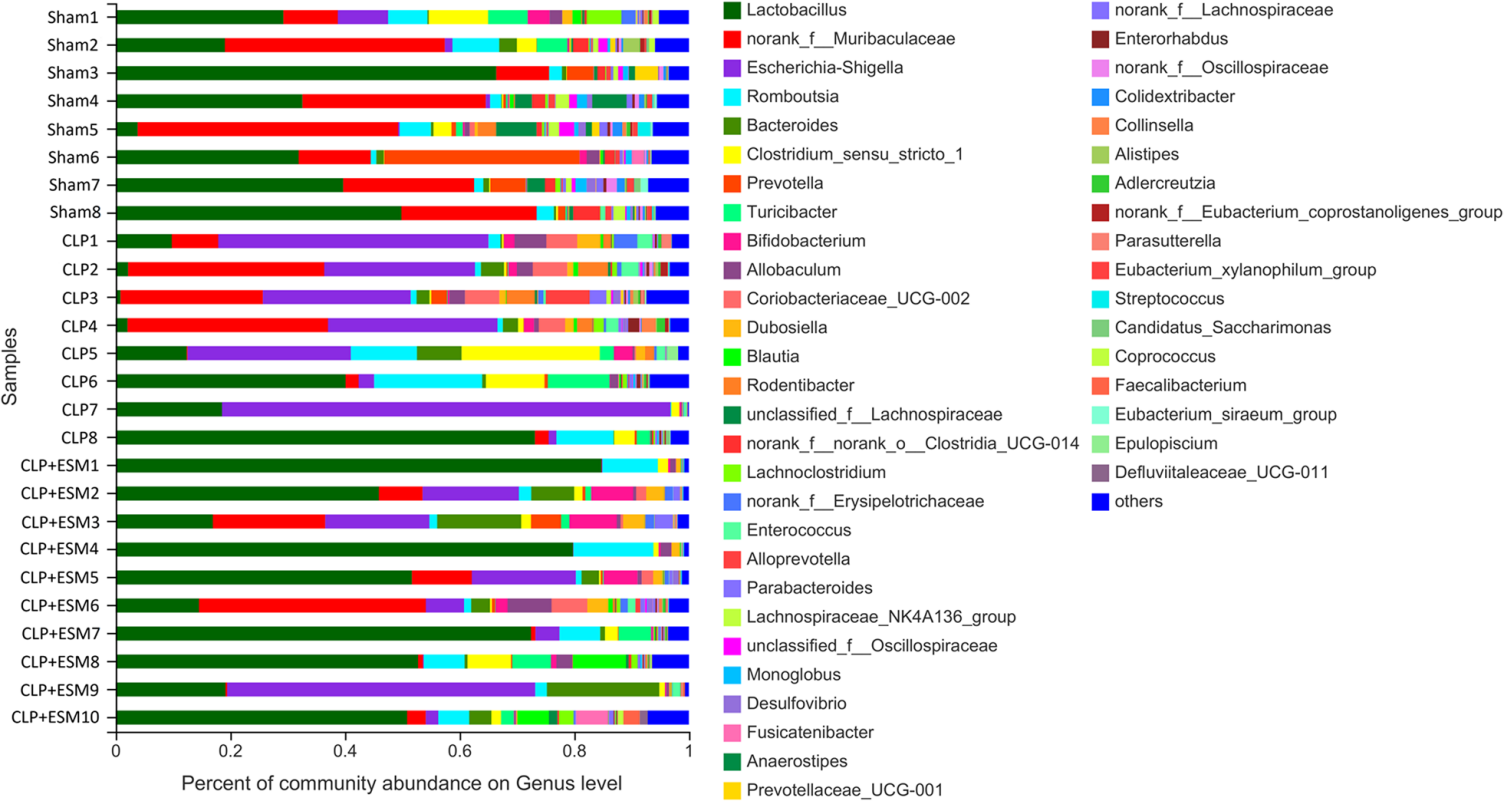
Relative abundance of bacterial taxa on genus level

**Fig. 5:**
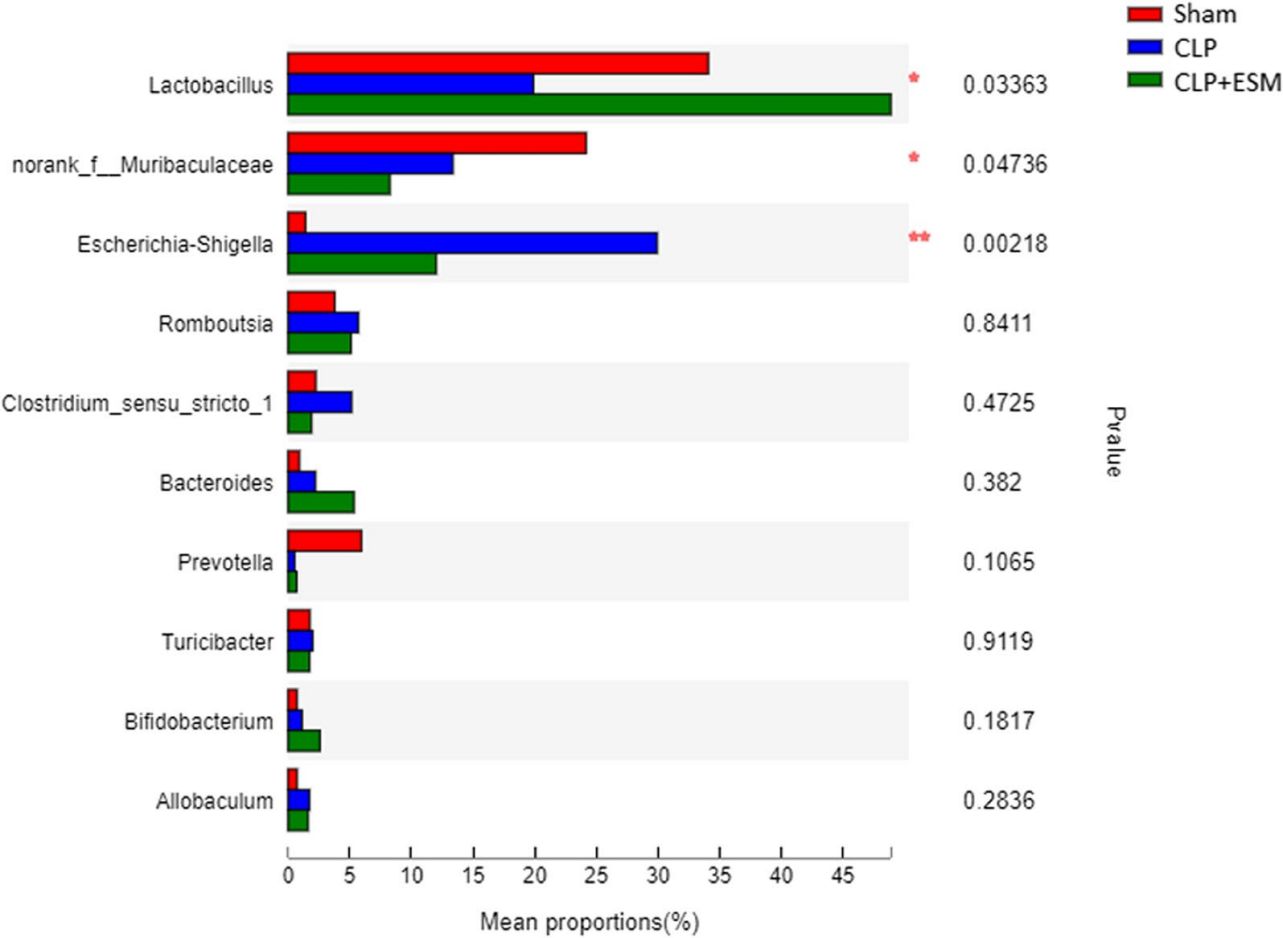
Abundance differences analysis (Wilcoxon rank-sum test) of gut microbiota community on genus level

**Fig. 6:**
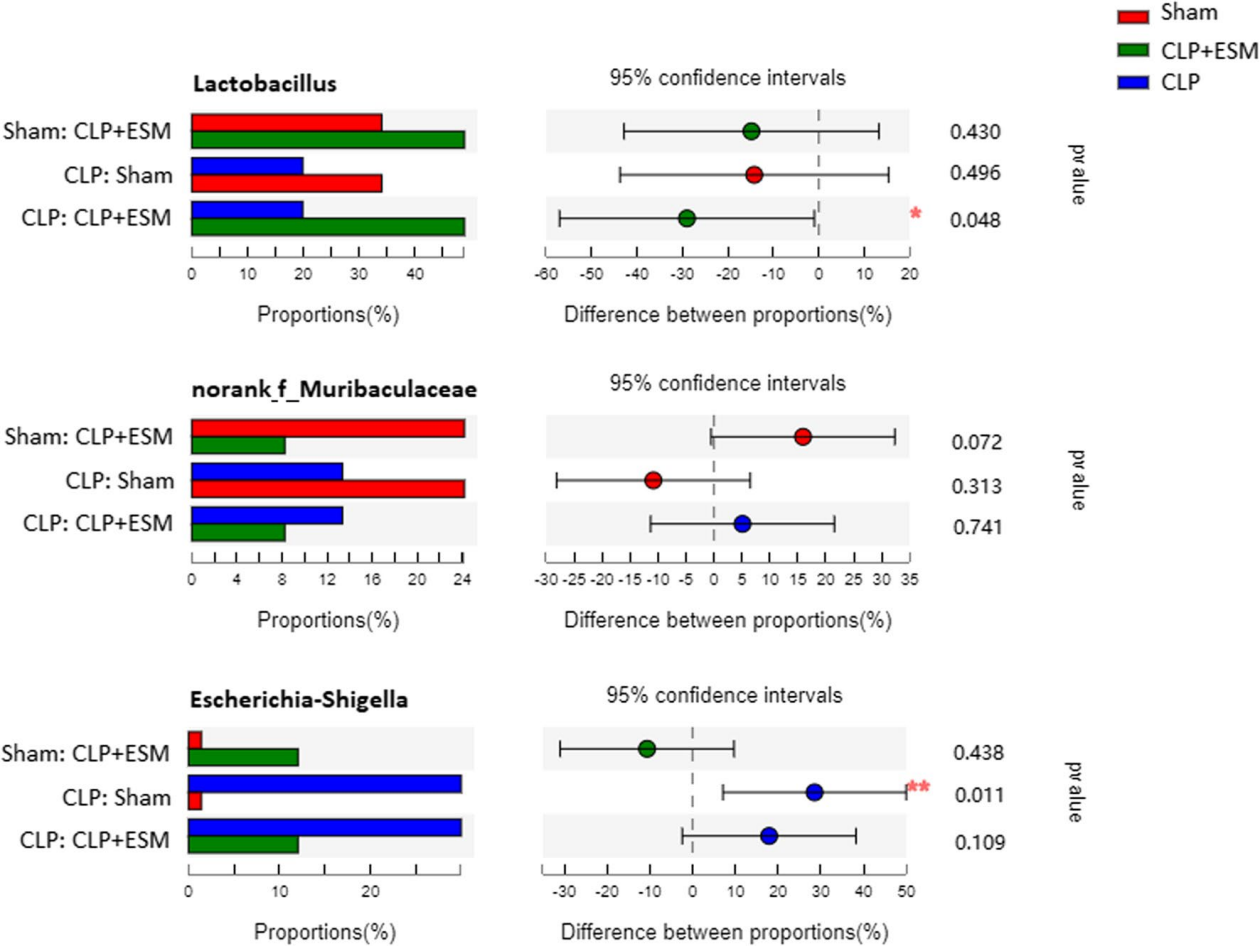
The top 3 taxa abundance with significant difference in genus level

The relative abundance of bacterial taxa at the genus level is shown in Fig. 4, and the top 10 taxa with significant differences in abundance at the genus level are shown in Fig. 5. Each pair of groups was further compared. Esmolol treatment marginally increased the abundance of *Lactobacillus* in rats after CLP surgery (*P* = 0.048). CLP surgery caused a large increase in the abundance of *Escherichia–Shigella* (*P* = 0.011). But there was no statistic difference between CLP group and CLP + ESM group in the abundance of *Escherichia–Shigella* (*P* = 0.109) (Fig. 6).

**Fig. 7:**
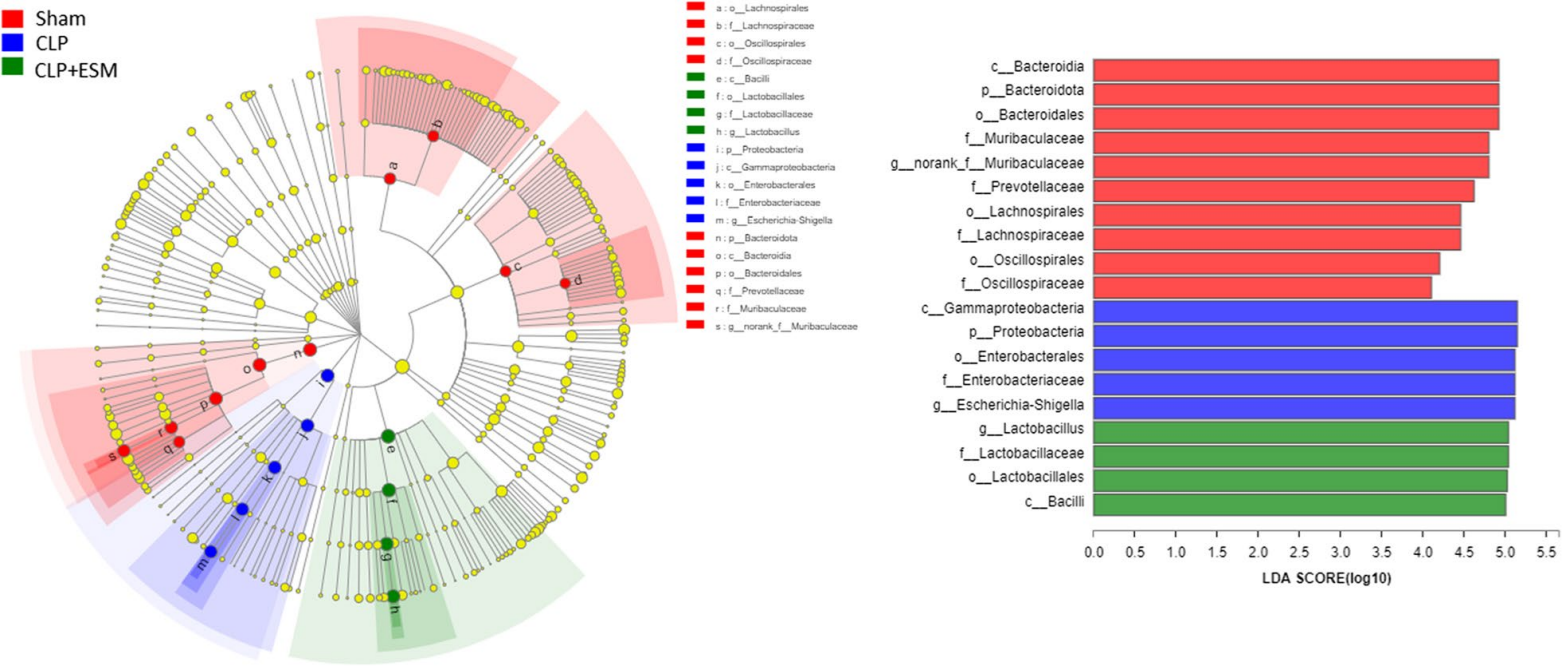
The plot cladogram and linear discriminant analysis (LDA) effect size (> 4) in the linear discriminant analysis effect size (LEfSe) analysis

LEfSe was used to identify the predominant bacteria. *Norank f Muribaculaceae*, *Escherichia–Shigella* and *Lactobacillus* were the predominant bacteria in the Sham group, CLP group and CLP + ESM group, respectively (Fig. 7).

### Effect of esmolol on the functional profiles of the gut microbiota

**Fig. 8:**
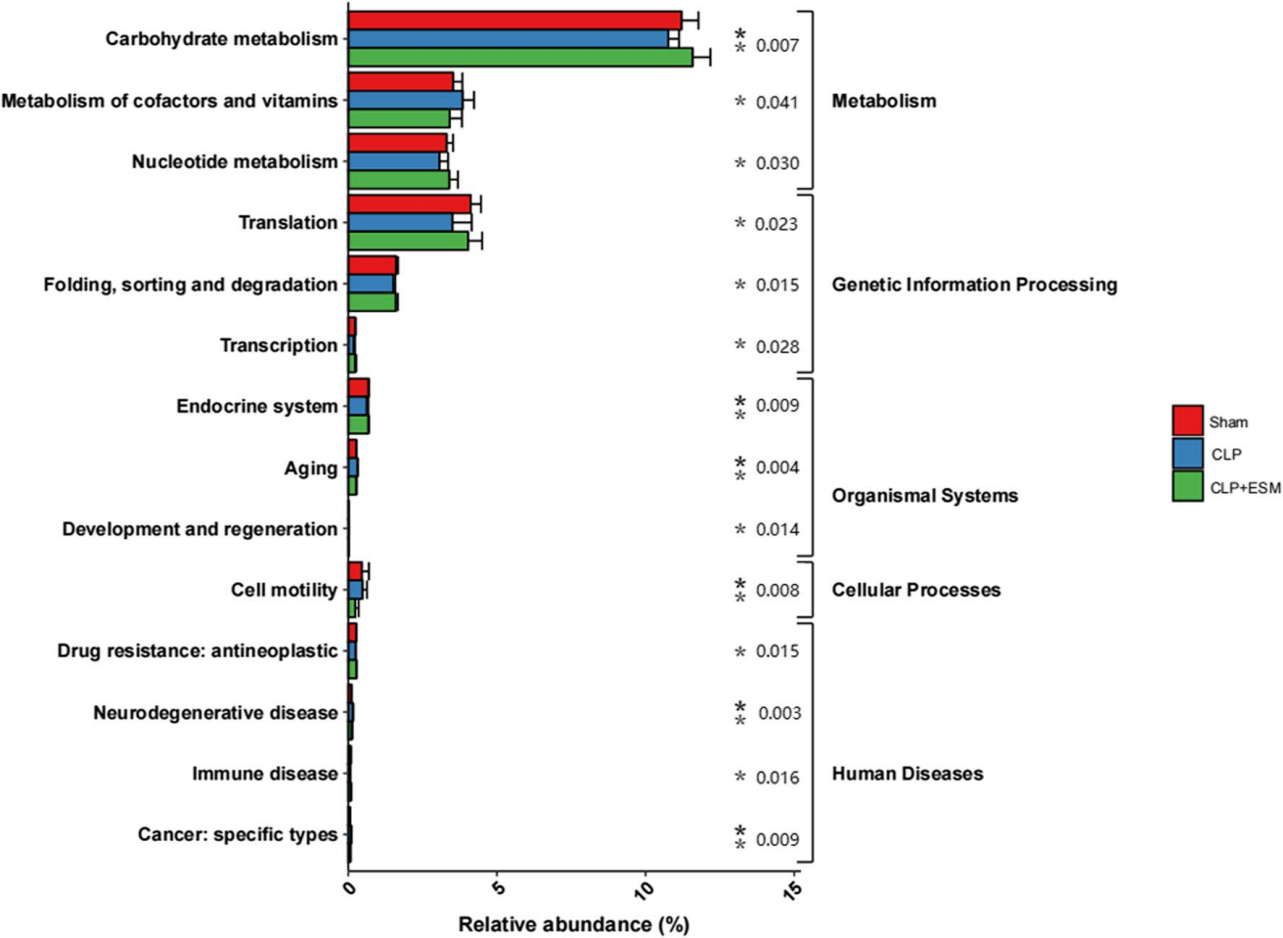
Predicted functional profiles of the gut microbiota

The significant differences in pathways (carbohydrate metabolism, metabolism of cofactors and vitamins, nucleotide metabolism, translation and so on) among these groups at level 2 of the KEGG pathway analysis are shown in Fig. 8. Compared to the CLP group, carbohydrate metabolism was significantly more enriched in the CLP + ESM group (*P* = 0.007).

### Effect of esmolol on intestinal edema, immunohistochemical staining of iNOS in colonocytes and nitric oxide concentration of colon feces

**Fig. 9:**
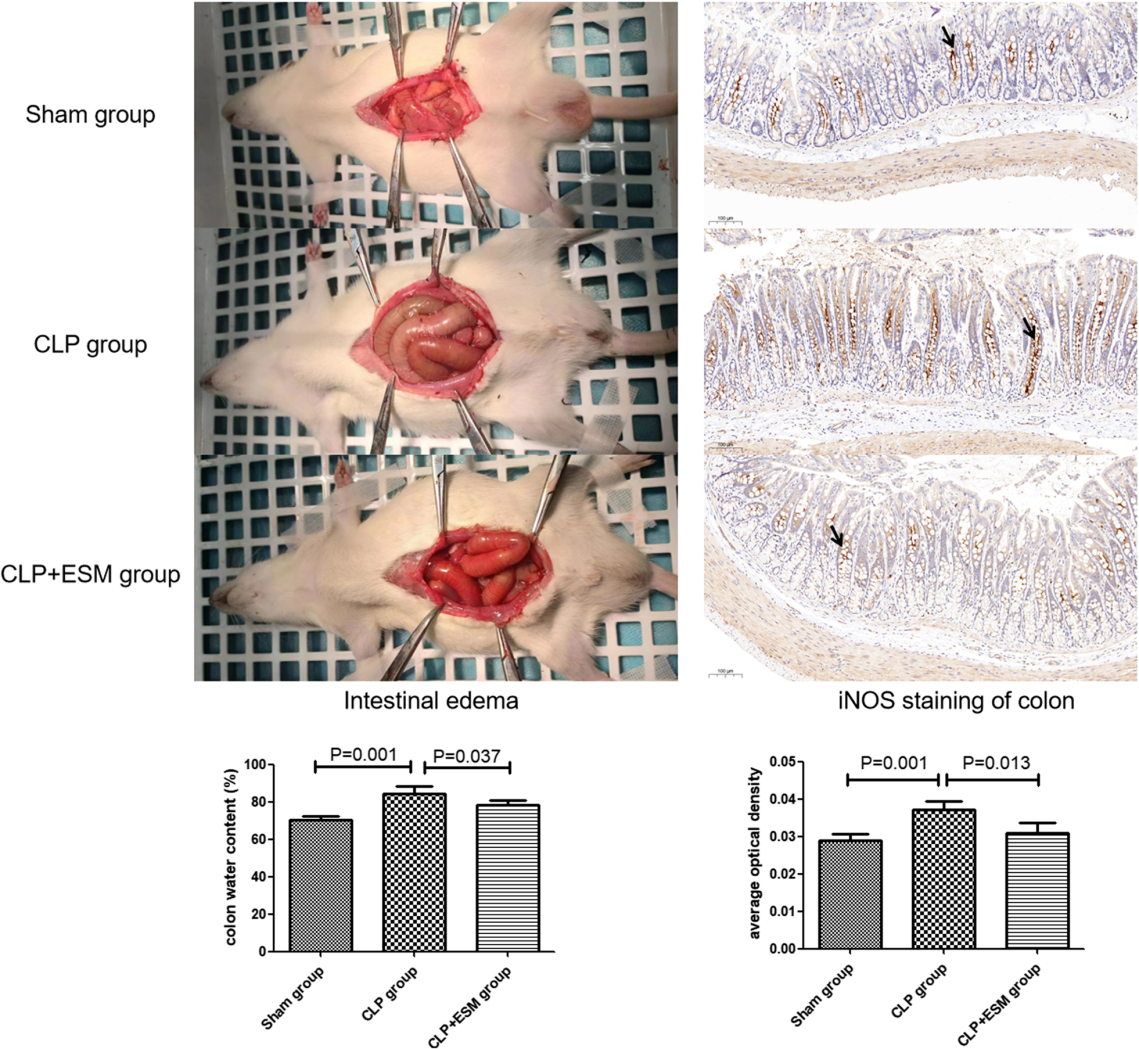
Intestinal edema and immunohistochemical staining of inducible nitric oxide synthase (iNOS) in colonocytes. (The iNOS expression area was stained by brown.)

CLP surgery increased the intestinal edema of rat, but esmolol treatment decreased the intestinal edema in the CLP rats (Fig. 9). In the immunohistochemical staining of iNOS in the colon, the iNOS expression in colonocytes stained by brown in the CLP group were much more than Sham group (*P* = 0.001). Compared to CLP group, the iNOS expression in colonocytes reduced after esmolol treatment (*P* = 0.013) (Fig. 9). The concentration of nitric oxide in colon feces was different in Sham group, CLP group and CLP + ESM group (1.31 ± 0.15μmmol/l vs. 1.98 ± 0.27μmmol/l vs. 1.51 ± 0.14μmmol/l, *P* = 0.001). In addition, the concentration of nitric oxide in CLP group was higher than Sham group (*P* = 0.001) or CLP + ESM group (*P* = 0.001).

## Discussion

In this study, esmolol had no significant effect on the α-diversity and β-diversity of the gut microbiota in the CLP rat model. However, it did influence the abundance of some bacteria, as it increased the abundance of *Lactobacillus*.

Esmolol is commonly used for treating tachycardia in sepsis without increasing adverse events, and it can even improve 28-day mortality for sepsis [17]. In our study, 4 rats died 24 h after CLP surgery, but only 2 rats undergoing esmolol treatment died. In addition to heart rate control, other effects were also found. Esmolol can alleviate dysfunction of gut microcirculation during sepsis [18]. Moreover, esmolol inhibited the inflammation by inhibiting the NF-κB-p6 pathway and apoptosis in gut tissue in a sepsis rat model [19]. There have been few studies on the influence of esmolol on the gut microbiota. In this study, we found that esmolol increased the abundance of *Lactobacillus*. In LEfSe analysis, *Lactobacillus* was the predominant bacteria in the CLP + ESM group. *Lactobacillus* is a kind of probiotic. In a murine sepsis model, *Lactobacillus* alleviated severe gut leakage, and reduced inflammatory responses and sepsis mortality [20]. In addition, *Lactobacillus* can attenuate stress-related disorders, such as anxiety and depression, through the regulation of γ-aminobutyric acid expression [21]. *Lactobacillus* supplementation also increased the amount of the short-chain fatty acid [22]. Decreases in short-chain fatty acids are associated with the disruption of the gut microbiota and sepsis. Therefore, the increase in abundance of *Lactobacillus* may be beneficial for sepsis. In the present study, we used PICRUSt to predict the functional profiles of the gut microbiota. We found that carbohydrate metabolism was significantly enriched in CLP rats after esmolol treatment. A previous study confirmed that *Lactobacillus* could improve carbohydrate metabolism [23, 24]. Wang Q et al. found that *Lactobacillus helveticus* R0052 increased fecal levels of galactose and maltose and decreased fecal levels of lactose and talose [23]. In addition, *Lactobacillus helveticus* R0052 could ferment lactose into easily absorbed lactic acid and provide additional nutrition for hosts [25].

Colonocytes can affect the microenvironment of the gut microbiome [11]. There are two opposing colonocyte phenotypes (C1 and C2). Proinflammatory signals, such as IFN-γ, can stimulate metabolic polarization into C1 colonocytes, which are characterized by high lactate release, low oxygen consumption, and elevated synthesis of iNOS. iNOS can generate nitric oxide, which can be converted into nitrate in the gut lumen. Nitrate can be used by facultative anaerobic Enterobacteriaceae (such as *Escherichia coli*). It also drives the expansion of facultative anaerobic bacteria, which has disadvantages for its competing microbes (obligate anaerobic microbes) [12]. Sepsis can increase the iNOS expression [13]. In our study, we also found the colonocytes with positive staining of iNOS and the nitric oxide concentration of colon feces increased in the CLP rat model. Moreover, the abundance of facultative anaerobic Enterobacteriaceae, such as *Escherichia–Shigella*, was increased in the CLP rat model. These findings can be explained by the above theory. In addition, it was confirmed that esmolol decreased the inflammation and iNOS expression in sepsis [13]. In our study, after esmolol treatment, the iNOS expression in colonocytes and the nitric oxide concentration of colon feces decreased. Moreover, the abundance of obligate anaerobic microbes, such as *Lactobacillus*, began to increase in the sepsis model after esmolol treatment. Therefore, we thought esmolol affect the gut microbiome by decreasing C1 colonocyte activation.

There are some limitations to this study. First, we selected the intraperitoneal injection of esmolol. In previous studies, continuous intravenous injection was the most common administration method. Therefore, we do not know whether the continuous intravenous injection has a similar effect as intraperitoneal injection. Second, esmolol may effect hemodynamic. Some hemodynamic parameters, such as blood pressure, were not monitored in the study. We cannot preclude the effect of hemodynamics on gut microbiome. Thirdly, because nitric oxide was extremely unstable, the nitric oxide level was estimated by the stable end-product of nitric oxide (the total nitrite and nitrate) concentration. Fourthly, antibiotic and analgesia were not used after CLP surgery in the study, which may reduce the correlation with the clinical condition of sepsis [26].

## Conclusion

In a rat of sepsis model, esmolol had no significant effect on the α-diversity and β-diversity of the gut microbiota. But it increased the fecal abundance of *Lactobacillus*. Moreover, esmolol reduced the iNOS expression in colonocytes and the nitric oxide concentration of colon feces.

## Data Availability

The datasets during the current study are available from the corresponding author on reasonable request.
